# Early-life stress affects Mongolian gerbil interactions with conspecific vocalizations in a sex-specific manner

**DOI:** 10.3389/fnbeh.2023.1128586

**Published:** 2023-05-10

**Authors:** Kate A. Hardy, Denise M. Hart, Merri J. Rosen

**Affiliations:** ^1^Department of Anatomy and Neurobiology, Hearing Research Group, Northeast Ohio Medical University, Rootstown, OH, United States; ^2^Department of Biological Sciences, Brain Health Research Institute, Kent State University, Kent, OH, United States

**Keywords:** early-life stress (ELS), auditory, communication, vocalizations, adolescence, sex differences, social behavior

## Abstract

During development, early-life stress (ELS) impairs cognition, learning, and emotional regulation, in part by disrupting neural circuitry in regions underlying these higher-order functions. In addition, our recent work indicates that ELS also alters simple sensory perception: ELS impaired auditory perception and neural encoding of short gaps in sounds, which are essential for vocal communication. The combination of higher-order and basic sensory disruption suggests that ELS is likely to affect both the perception and interpretation of communication signals. We tested this hypothesis by measuring behavioral responses to conspecific vocalizations (those emitted by other gerbils) in ELS and untreated Mongolian gerbils. Because stress effects often differ by sex, we separately examined females and males. To induce ELS, pups were intermittently maternally separated and restrained from post-natal days (P) 9–24, a time window when the auditory cortex is most sensitive to external disruption. We measured the approach responses of juvenile (P31–32) gerbils to two types of conspecific vocalizations: an alarm call, which is emitted to alert other gerbils of a potential threat, and the prosocial contact call, which is emitted near familiar gerbils, especially after separation. Control males, Control females, and ELS females approached a speaker emitting pre-recorded alarm calls, while ELS males avoided this source, suggesting that ELS affects the response to alarm calls in male gerbils. During playback of the pre-recorded contact call, Control females and ELS males avoided the sound source, while Control males neither approached nor avoided, and ELS females approached the sound. These differences cannot be accounted for by changes in locomotion or baseline arousal. However, ELS gerbils slept more during playback, suggesting that ELS may reduce arousal during vocalization playback. Further, male gerbils made more errors than females on a measure of working memory, but the sex difference of cognition in this context may stem from novelty aversion rather than impaired memory. These data indicate that ELS influences behavioral responses to ethologically relevant communication sounds in a sex-specific manner, and are among the first to demonstrate an altered response to auditory stimuli following ELS. Such changes may arise from differences in auditory perception, cognition, or a combination of factors, and suggest that ELS may affect auditory communication in human adolescents.

## 1. Introduction

Experiences during early development have lasting impacts on individuals (Eiland and Romeo, 2013). The experience of early-life stress (ELS) has been studied particularly well regarding its effects on the prefrontal cortex (PFC), hippocampus, and amygdala, and the behaviors those regions govern, such as cognition, anxiety, and memory (Rainnie et al., 2004; Liston et al., 2006; Ivy et al., 2010). Research into the effects of ELS on the auditory system, however, has been limited to sensory gating and detection of small gaps in sound (Ellenbroek et al., 2004; Ye et al., 2022). Chronic stress affects the auditory system in *adult* rats, by inducing dendritic atrophy and impairing behavioral measures such as auditory attention and auditory conditioned avoidance (Dagnino-Subiabre et al., 2005; Pérez et al., 2013). Thus, it follows that chronic stress during development may have an even greater, and longer lasting, impact on auditory processing. Previous findings (Caras and Sanes, 2015; Mowery et al., 2015; Green et al., 2017) corroborate that manipulations of experiences during early post-natal development have effects that are clearly detectable in adolescence, including impaired perception of temporally-varying signals.

Temporal processing (i.e., the ability to detect small changes in sound over time) is especially vulnerable to modification by experience because of a period of maturation that extends into adolescence (Chang et al., 2005; Rosen et al., 2010; Sanes and Woolley, 2011). Because accurate temporal processing is required to comprehend the meaning of communication sounds, altered temporal processing may lead to differential processing of vocalizations (Drullman et al., 1994; Ter-Mikaelian et al., 2013; Eggermont, 2015). Vocal communication serves an important role in the survival of countless species by enabling animals to work cooperatively, identify individuals, recognize isolation calls of offspring, increase eating efficiency by reducing vigilance, and decrease likelihood of becoming prey (Brudzynski, 2010; Chaby et al., 2016).

A recent study from our lab has revealed that ELS in the Mongolian gerbil impairs temporal processing in adolescence, which suggests that vocalization perception may also be impaired (Ye et al., 2022). Consistent with such an ELS-induced temporal processing deficit, children raised in low socioeconomic status (SES) environments, a common proxy for ELS in humans, have increased risk for impaired speech perception (Benasich and Tallal, 2002). In addition, low SES is associated with unstable auditory brainstem responses, impaired auditory attention, reduced surface area of cortical language regions, and impaired phonetic discrimination ability in children (D’Angiulli et al., 2008; Skoe et al., 2013; Conant et al., 2017; McDermott et al., 2019). However, it has not been explicitly tested whether ELS alters behavioral responses to communication signals.

Studying ELS-induced effects requires separately examining males and females because ELS confers differential changes based on sex, sometimes in completely opposing directions (Luine et al., 2017). For example, ELS leads to depressive-like symptoms in female but not male mice (Goodwill et al., 2019). Further, during adolescence, temporal discrimination abilities mature earlier in males than females (Huyck and Wright, 2017), suggesting differential susceptibility to developmental stress effects.

Sex differences are also apparent in interpretation of some, but not all, vocalizations in adult mice. Ultrasonic vocalizations emitted during friendly greetings and sexual encounters cause an increase in risk-assessment behaviors in female, but not male mice, suggesting that the sexes perceive the valence (i.e., pleasantness or unpleasantness) of this type of vocalization differently. However, playback of a distress call caused *both* male and female mice to increase risk-assessment behaviors (Ghasemahmad et al., 2022). Thus, the sexes perceive the emotional content of some vocalizations differently. Because this is not true for all vocalizations, it is important to utilize call types with both positive and negative valence. Multiple contexts increase the likelihood of uncovering important sex-related behavior differences. Using both positive and negative call types is especially important for studies of ELS, as ELS alters neural regions responsible for processing emotions, namely, the PFC and amygdala (Cardinal et al., 2002; van Bodegom et al., 2017; Burgdorf et al., 2020).

Here, we tested the effects of ELS and sex in an ethologically relevant design that allowed highly vocal Mongolian gerbils the freedom to choose whether, and to what degree, to engage with a conspecific vocalization (Yapa, 1994; Kobayasi and Riquimaroux, 2012). Gerbils were placed in a Y-maze and their behavior was recorded in response to vocalization playback. Two vocalization types of polar valence, a contact call and an alarm call, were chosen to test the hypothesis that ELS or sex may cause valence-specific outcomes. Though ELS did not affect general locomotion or exploration, it did affect interest in the vocalizations differentially based on sex and valence. These findings, along with our lab’s recently published work (Ye et al., 2022), suggest that in adolescent Mongolian gerbils, ELS affects the perception of communication signals, potentially by disrupting temporal processing, which is necessary to comprehend vocal communication (Drullman et al., 1994; Ter-Mikaelian et al., 2013). Such an impairment in human adolescents may be detrimental in multiple aspects of their lives such as academic performance and social interactions (Gravel et al., 1995; Landerl and Willburger, 2010).

## 2. Materials and methods

### 2.1. Subjects

All procedures relating to the maintenance and use of animals in this study were approved by the Institutional Care and Use Committee at Northeast Ohio Medical University (NEOMED). Forty-one Mongolian gerbils (*Meriones unguiculatus*) from multiple litters were raised by internally bred pairs. Lineage of the breeding pairs originated from Charles River Laboratories Inc. (Wilmington, MA, USA). Upon weaning at post-natal day (P) 30, females (*n* = 19) and males (*n* = 22) were housed as 2–5 same-sex littermates in a 12 h light/dark cycle (light cycle: 7:00–19:00 h) with food and water *ad libitum*. Experiments took place during the light cycle between 9:00 and 15:00 h, as gerbils are crepuscular (active throughout the day, most active at dawn and dusk). Two treatment groups included ELS animals (*n* = 20) and age-matched, non-ELS animals (“Control,” *n* = 21).

### 2.2. ELS induction

**FIGURE 1:**
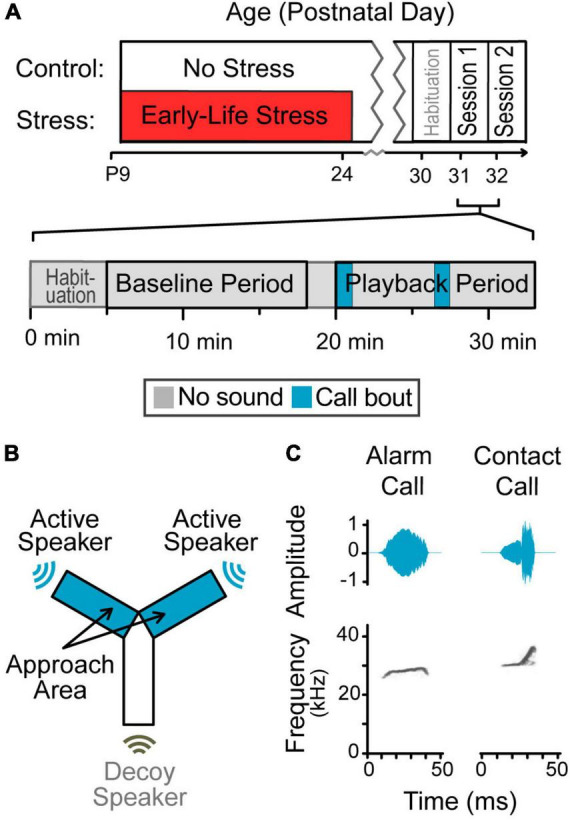
Design of the playback study. **(A)** Experimental timeline. Animals were randomly assigned to one of two groups. Half of the animals experienced early-life stress during a time window encompassing maturation of the auditory cortex, and half did not. All animals were tested with one of two stimuli during adolescence, counter balanced. Session timeline. A total of 13 min segments during silent baseline and vocalization playback were used for the analysis. **(B)** Y-maze schematic. Gerbils roamed freely during a period of silence followed by a period of vocalization playback, in a custom-built Y-maze designed to minimize sound reflections. Sound only emerged from two of the three arms (blue); the last arm had a non-functioning speaker (uncolored). **(C)** Amplitude envelopes and spectrogram of exemplar syllables. Vocalization playback included these syllables and eight others concatenated into bouts.

Early-life stress was induced by unpredictable, intermittent maternal separation and restraint for 2 h, on 10 non-consecutive days between P9 and P24. This is an age at which there is high neural plasticity in the auditory cortex (Figure 1A; Mowery et al., 2015). Unpredictability of stressors is a crucial contributor to stress-related behavioral changes (Bath et al., 2017b). Pups were removed from their home enclosure and transferred to a separate room before being placed in a plastic, cylindrical restraining device. Each restraining device was then placed in a small sound-attenuating box such that no pup could see, hear, or smell any other pups. During this time, Control animals remained undisturbed in their home enclosure with both parents. A previous study in our lab confirmed this procedure induces stress based on altered corticosterone levels and responses to startling stimuli (Ye et al., 2022).

### 2.3. Testing apparatus

A custom-built, Y-shaped maze was designed to minimize sound reflections (Figure 1B). The walls were created from ¼” vertical acrylic rods placed 15 mm apart, surrounded by sound-attenuating foam. Arms were placed at 120^°^ angles and measured 10 cm wide, 40 cm long, and 30 cm high. Active speakers were located outside of two of the arms and a decoy speaker was located outside the third arm. Testing took place inside a single-walled booth (152 cm wide, 122 cm length, and 200 cm high; Industrial Acoustics, Bronx, NY, USA). The Y-maze was placed on a table underneath a digital camera (Logitech 270C, Lausanne, Switzerland) that recorded each session for analysis offline.

### 2.4. Auditory stimuli

#### 2.4.1. Vocalization recording

Multiple unrelated groups of gerbils were used in vocalization recordings. Each home enclosure was placed on a table in the booth described above with an ultrasonic microphone (UltraSoundGate 116, Avisoft Bioacoustics, Glienicke, Germany) 35 cm above the enclosure. Calls were recorded onto a PC running Windows 10 with Avisoft RECORDER software (Avisoft Bioacoustics, Glienicke, Germany; sampling rate 250 kHz, 16-bit resolution). The groups were either same-sex, adult cage-mates or families (i.e., male and female parents and 2–4 juvenile offspring). Contact and alarm calls were identified by previously reported spectro-temporal features described below (Yapa, 1994; Kobayasi and Riquimaroux, 2012; Ter-Mikaelian et al., 2012).

Gerbils emit alarm calls to alert the colony about threats, such as the presence of predators, and during fighting behavior; these are considered to have negative valence (Yapa, 1994; Kobayasi and Riquimaroux, 2012). This less common call is characterized as an arched frequency modulated call (mean characteristics reported by Ter-Mikaelian et al. (2012): frequency 23.3 ± 1.7 kHz, syllable duration 116 ± 69 ms, bandwidth 2.8 ± 2.0 kHz) (Figure 1C). Alarm calls were generally elicited by startling the gerbils, but two alarm call exemplars used in stimulus playback were produced with no external manipulation and no changes in environment detected by the researcher.

Gerbils emit contact calls when in proximity to one or more conspecifics; these are considered affiliative with a positive valence (Kobayasi and Riquimaroux, 2012). This common call is characterized as a short, upward frequency-modulated call (mean frequency 30.1 ± 2.4 kHz, syllable duration 26 ± 8 ms, bandwidth 4.1 ± 2.2 kHz) (Figure 1C). Contact calls were elicited by separating then reuniting cage-mates after various lengths of time (5 min to 2 h). A variety of durations were chosen, in case signals differed across durations. Although potential differences were not analyzed for this study, the stimulus used was assembled from across this range.

#### 2.4.2. Vocalization playback

A single playback sequence was created for each alarm and contact vocalization category; these are openly available at https://osf.io/6pszq/?view_only=88fd644de88041a8945c18902f578f75. Both tracks contained nine exemplars, randomly concatenated, to create two vocalization bouts of 45–75 s. The bouts were separated by 330–390 s (durations randomized) to allow for a time window of analysis encompassing the bouts and extending for several minutes (see section “2.6 Behavioral analysis”).

Contact calls often occur in rapid succession (median inter-syllable-interval, ISI: 180 ms) while alarm calls typically occur at a much slower rate (median ISI: 893 ms) or as a single syllable. Because eight of the nine alarm exemplars recorded for this study were single syllables, bouts used a 500–1,000 ms ISI, increasing the natural contact ISI and decreasing the alarm ISI, to equate stimulus presentation across conditions. The pre-recorded vocalization bouts were high-pass filtered at 2,000 Hz and presented at 80 dB SPL (peak amplitudes) at 2” from the speaker and 62 dB SPL in the center of the maze.

Stimuli were calibrated and presented in free-field, from a MF1 multi-field magnetic speaker (Tucker-Davis Technologies, Alachua, FL, USA) through a PC running Windows 10, using Adobe Audition 3.0 software (San Jose, CA, USA). Calibration data were collected using a ¼inch free-field microphone [Brüel and Kjær (B and K) model 4,939] placed two inches from the speaker. Frequency sweeps at 0.5–30 kHz were presented and recorded using custom-written MATLAB (Mathworks, Natick, MA, USA) scripts (S. J. Shanbhag). The microphone output was amplified (B&K 2,690-A Nexus conditioning amplifier) and digitized at a sampling rate of 195.3 kHz. Resulting calibration files were applied to each vocalization stimulus file to spectrally flatten the speaker output, ensuring similar presentation levels across frequencies.

### 2.5. Experimental design

Gerbils were habituated to the testing room at age P30, and testing commenced at age P31. This age was chosen to assess differences in responses to vocalizations during early adolescence because ELS in gerbils has been demonstrated to affect encoding and perception of temporally-varying auditory signals at this age (Ye et al., 2022). Testing required two sessions in as many days. Alarm calls were presented during one session and contact calls during the other session, counterbalanced across groups for which vocal type occurred first. Because olfactory cues may be necessary for response to playback vocalizations, the end of each arm contained material with appetitive odors (i.e., fresh urine from same-sex cage-mate) (Farrell and Alberts, 2002).

The test started when a gerbil was placed in the center of a Y-maze and allowed to roam freely. After a 20 min silent baseline period (Kobayasi et al., 2014), vocalizations emerged from the ends of two arms non-simultaneously, alternating semi-randomly (Figure 1B). These two arms are the “Approach Arms,” where the gerbil can be near the sound source. The orientation of the approach arms within the room was randomized for each session.

### 2.6. Behavioral analysis

The gerbil’s location within the maze was analyzed offline with DataWave SciWorks VideoBench (DataWave Technologies, Inc., Parsippany, NJ, USA). Time spent in each location was measured during a 13 min period during each baseline and playback. This time window is sufficient to present two bouts of vocalizations and encompass an extended period of behavioral alertness evoked by stimuli (Randall et al., 2002). For this reason, many vocalization playback studies measure behavior for >3 min after stimulus playback (Wöhr and Schwarting, 2007; Chabout et al., 2012; Grimsley et al., 2013; Fernández-Vargas and Johnston, 2015).

Data collection for baseline began after 5 min to reduce likelihood of novelty-induced anxiety when the gerbils were first placed into the maze.

#### 2.6.1. Locomotion

As there may be group differences in locomotion or motivation, which could influence the response to the vocalizations, we quantified the arm entry rates during baseline and playback as the average number of entries per minute awake.

#### 2.6.2. Alternation rate

As a proxy for working memory (one aspect of cognition), we measured each gerbil’s arm entrance pattern. A low number of returns to a recently visited arm requires that the gerbil remembers which arm(s) it was in most recently and chooses to explore the least recently visited arm. We recorded the number of times an animal reentered the most recent or second most recent arms (i.e., did not enter the least recently visited arm) then divided that number by the total number of arm entrances to account for possible individual differences in activity levels.


$$A\,l\,t\,e\,r\,n\,a\,t\,i\,o\,n\,e\,r\,r\,o\,r\,r\,a\,t\,e=\frac{N\,u\,m\,b\,e\,r\,o\,f\,r\,e\,p\,e\,a\,t\,e\,d\,a\,l\,t\,e\,r\,n\,a\,t\,i\,o\,n\,s}{N\,u\,m\,b\,e\,r\,o\,f\,s\,u\,c\,c\,e\,s\,s\,f\,u\,l\,a\,l\,t\,e\,r\,n\,a\,t\,i\,o\,n\,s-2}$$

A second measure of arm visitation pattern was used to determine whether gerbils showed a preference for entering more- or less-recently visited arms. We recorded the number of times an animal entered an arm different from the previous two arms and divided that number by total arm entrances to get a spontaneous alternation rate. A rate of 0.5 indicates the animals either do not remember or do not show a preference for more- or less-familiar arms. A rate closer to 1 indicates the animals prefer to explore less-familiar arms, and a rate closer to 0 indicates a preference for entering more-familiar arms.

$$S\,p\,o\,n\,t\,a\,n\,e\,o\,u\,s\,a\,l\,t\,e\,r\,n\,a\,t\,i\,o\,n\,r\,a\,t\,e=\frac{N\,u\,m\,b\,e\,r\,o\,f\,f\,u\,l\,l\,a\,l\,t\,e\,r\,n\,a\,t\,i\,o\,n\,s}{N\,u\,m\,b\,e\,r\,o\,f\,t\,o\,t\,a\,l\,a\,r\,m\,e\,n\,t\,r\,i\,e\,s-2}$$

#### 2.6.3. Sleep

A gerbil was presumed to be sleeping when it adopted a head-tucked position (standing on rear legs with head bent down, tucked between rear legs) and remained motionless for at least 10 s.

#### 2.6.4. Vocalization approach

Because animals differed greatly in amount of time they spent awake, and therefore able to interact with their environment, we excluded time the gerbil was asleep (except when time spent sleeping was the dependent variable). Thus, we analyzed vocalization approach as a proportion of total time awake in the session. Further, it is crucial to normalize the approach proportion during playback to the approach proportion during pre-playback baseline to account for possible bias from extra-maze cues. Our normalized proportion of time spent approaching the vocalizations was therefore calculated as:

$$A\,p\,p\,r\,o\,a\,c\,h\,m\,e\,t\,r\,i\,c=\frac{\begin{array}{c} P\,l\,a\,y\,b\,a\,c\,k\,p\,r\,o\,p\,o\,r\,t\,i\,o\,n-B\,a\,s\,e\,l\,i\,n\,e\,p\,r\,o\,p\,o\,r\,t\,i\,o\,n \end{array}}{B\,a\,s\,e\,l\,i\,n\,e\,p\,r\,o\,p\,o\,r\,t\,i\,o\,n}$$

### 2.7. Statistics

Results were analyzed using SPSS Statistics for Windows, version 18.0 (SPSS Inc., Chicago, IL, USA) by a repeated measures analysis of variance (ANOVA). Data were assessed for normality using the Shapiro-Wilk test. If the data failed to meet the assumption of normality, data were transformed using the Yeo-Johnson method, an extension of the Box-Cox power transform that can be applied to positive and negative data (Peltier et al., 1998; Yeo and Johnson, 2000).

We used paired samples *t*-tests exclusively for within-subject comparisons (habituation vs. playback and alarm vs. contact). We used one-way ANOVAs for between-group effects (treatment, sex). We prefer this *post-hoc* test to the independent samples *t*-test because ANOVAs produce an effect size (ηp^2^) and the rates of significance do not differ between the tests. The effect sizes are reported for all significant one-way ANOVAs.

When needed, tests were corrected for multiple comparisons using the Benjamini-Hochberg procedure (false discovery rate: 0.1, Benjamini and Hochberg, 1995; Mancera et al., 2018; Saiz-Alía et al., 2019) unless no statistical significance was found for that test. With this procedure, *p*-values remain unadjusted, and differences are statistically significant only when the original *p*-value is less than the Benjamini-Hochberg critical value.

Data are presented as mean ± standard error of the mean (SEM), and statistical significance was determined as *p* < 0.05. In violin plots, box edges are 25th and 75th percentiles, the median is an empty circle, and lines extend to the edge of the kernel density plot are the most extreme data points.

One animal was removed from the study because of health concerns and another was removed because of experimental error. Two sessions for one animal were excluded because of a corrupted video file. One session each from five animals was excluded from all tests except time spent sleeping, because they were awake for less than 10% of the 13 min playback sample period.

## 3. Results

### 3.1. Baseline locomotion

**FIGURE 2:**
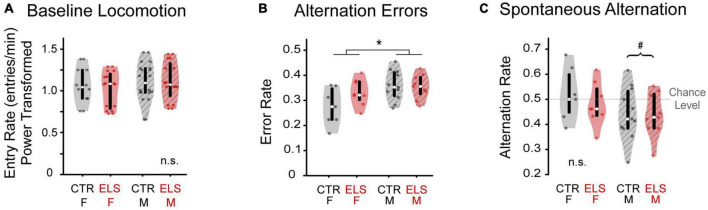
Early-life stress treatment does not affect locomotion or cognition. Sex influences cognition, but not locomotion. **(A)** Number of arm entries per minute was calculated during the silent baseline. There was no difference in locomotion between early life stress (ELS) (red) and Control gerbils (gray), nor between female (solid) and male gerbils (striped). **(B)** Female and male gerbils performed differently in one measure of cognition. The alternation error rate was measured as the number of times an animal returned to either of the previous two arms, normalized to total arm entries. ELS and Control animals did not differ in arm return rate. Female gerbils made fewer errors than males. **(C)** Spontaneous alternation was calculated as the number of sequential entries of the arms without repeats, normalized to total arm entries. While female gerbils performed at chance level of 0.5, male gerbils performed significantly worse than chance. Box edges are the interquartile range, with whiskers extending to minimum and maximum values and median horizontal line. *Significant at *p* < 0.05. ^#^Significant deviation from chance.

To interpret whether early-life stress (ELS) affected the amount of time that animals spent near the vocalizations, a necessary first step was to assess whether either ELS treatment or sex affected locomotion around the Y-maze. We measured the number of total arm entries during 13 min of the pre-playback silent baseline, starting after a 5 min delay (Figure 2A). We did not expect a difference in treatment groups but predicted male animals might be more mobile, given their role in the colony is to patrol the borders of the territory (Agren et al., 1989a).

The total number of arm entries was divided by the number of seconds awake and multiplied by 60 to get the locomotion rate as number of arm entries per minute. Two animals were excluded because they had fewer than four arm entries during baseline or playback. Because these data were not normally distributed, we applied a Yeo-Johnson power transformation. No differences in locomotion were found for treatment [Figure 2A, ANOVA: F_(1, 31)_ = 0.055, *p* > 0.20] or sex [F_(1, 31)_ = 0.653, *p* > 0.20], nor were there any interaction effects [F_(1, 31)_ = 0.003, *p* > 0.20]. The similarity in locomotion in both Control and ELS animals removes locomotion as a potential confound in interpreting time spent near vocalizations during playback.

### 3.2. Baseline alternation rate

To best interpret any effects of early-life stress (ELS) on vocal preference, we first examined several elements of gerbil’s behavior in the Y-maze during silence in baseline. We quantified the sequence of arm entries as alternation error rate and spontaneous alternation rate. Spontaneous alternation is the sequential entry of the arms without repeats, and is a measure of working memory, which could potentially be affected by ELS. Alternation errors are a failure to spontaneously alternate. Based on the findings in a similar study of ELS in adolescent rats (Llorente et al., 2011), we did not expect to see any effect of ELS on alternation error (AE) rate. As predicted, ELS and Control animals did not differ in the AE rate [Figure 2B, ANOVA, no main effect of treatment: F_(1,34)_ = 1.748, *p* = 0.196]. We found that female gerbils made fewer errors than males [female: 0.303 ± 0.017, male: 0.355 ± 0.012; ANOVA, main effect of sex: F_(1,34)_ = 7.122, *p* = 0.012, ηp^2^ = 0.187], which indicates that male, more than female, gerbils returned to recently visited arms while exploring in the Y-maze. There was no interaction effect of treatment by sex [F_(1,34)_ = 2.333, *p* = 0.137] although Control females did tend to make fewer errors than ELS females [Control female: 0.273 ± 0.073, ELS female: 0.330 ± 0.053; one-way ANOVA: *F*_(1,13)_ = 3.046, *p* = 0.105, ηp^2^ = 0.187]. There was no such trend in male gerbils [Control male: 0.356 ± 0.018, ELS male: 0.352 ± 0.019; *F*_(1,18)_ = 0.028, *p* > 0.20].

Spontaneous alternation (SA) rate was then determined from each entrance to an arm different from the previous two arms visited. SA rate of ELS gerbils did not differ from Controls [Figure 2C, ANOVA: F_(1,34)_ = 0.496, *p* > 0.20]. There was a trend toward female gerbils having a greater SA rate than males [Female: 0.499 ± 0.024; Male: 0.442 ± 0.021, F_(1,34)_ = 3.191, *p* = 0.084, ηp^2^ = 0.093]. There was also no interaction between treatment and sex [Female CTR: 0.520 ± 0.102, Female ELS: 0.480 ± 0.085, Male CTR: 0.445 ± 0.104, Male ELS: 0.438 ± 0.087, F_(1,34)_ = 0.256, *p* > 0.20].

Spontaneous alternation rates across all animals approached a near significant difference from chance level of 0.5 [Figure 2C, 0.466 ± 0.096, one-sample *t*-test, t_(1,34)_ = –2.080, *p* = 0.045, but *p* > BH critical value: 0.033]. Further investigation of the near-significant effect of sex on SA rate, revealed that females perform at chance level [0.499 ± 0.092, t_(1,14)_ = –0.061, *p* > 0.20], while males show SA rates significantly below chance [0.442 ± 0.094, t_(1,19)_ = 2.754, *p* = 0.013, *p* < BH critical value: 0.014]. This suggests that male gerbils preferred to re-enter a recently explored arm over exploring a less familiar arm.

Taken together, these results suggest that the ELS and Control animals have similar cognitive abilities and that this is not likely to affect other measurements. Sex, however, may influence other outcomes as females performed at chance level, while males performed below chance and may be averse to exploring less-familiar areas.

### 3.3. Time spent sleeping

When placed in a stressful environment, such as a new location, arousal is typically increased. High arousal is also a defining characteristic of anxiety and is heightened by stress (De La Casa et al., 2016). We therefore used the amount of time spent sleeping as a proxy for arousal, which may reflect anxiety-like behaviors. While no differences for treatment or sex were anticipated, any differences in baseline arousal and anxiety-like behaviors in response to the vocalization playback must be ruled out to assist interpretation of the remaining data.

**FIGURE 3:**
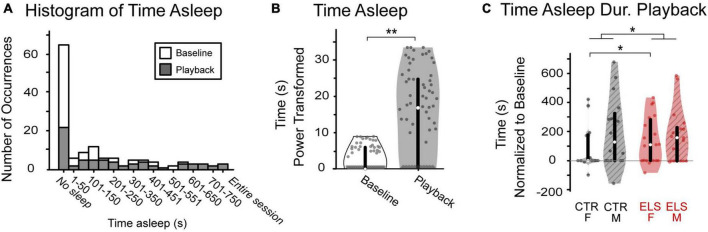
Vocalization playback and early life stress (ELS) treatment affect level of arousal, an anxiety-related behavior. **(A)** Occurrences of sleeping varied greatly across animals. While most gerbils tended to not sleep during either period, data were skewed right with some gerbils sleeping the entire session. **(B)** There was a significant increase in amount of time spent sleeping from baseline (white) to playback (gray). **(C)** ELS, but not sex, affected sleeping during playback, normalized to baseline. ELS animals (red) spent significantly more time sleeping during playback than Control. Box edges are the interquartile range, with whiskers extending to minimum and maximum values and median horizontal line. *Significant at *p* < 0.05. **Significant at *p* < 0.001.

The amount of time spent sleeping varied greatly across animals. While most gerbils tended to not sleep during either period, data were skewed right with some gerbils sleeping the entire session (Figure 3A). Regardless of treatment and sex, all animals spent less time sleeping during baseline than during playback, as anticipated given the length of our experiment, with a 20 min baseline immediately preceding playback [Figure 3B, Baseline: 2.703 ± 0.290 s, Playback: 14.750 ± 1.059 s, paired-samples *t*-test, t_(1,133)_ = –12.433, *p* < 0.001]. The valence of the stimulus may affect level of arousal; therefore, we first compared the amount of time spent sleeping during each playback period and found no difference. Because of their similarity, we combined stimulus types in subsequent sleep analyses [paired-samples *t*-test, t_(1,31)_ = 1.309, *p* > 0.20].

We then compared treatment, sex, and treatment by sex effects on time spent sleeping normalized to an individual’s baseline (time spent sleeping during playback minus baseline). Sex did not influence the amount of time spent sleeping during playback, normalized [Figure 3C, ANOVA: F_(1,131)_ = 1.236, *p* > 0.20]. There was a significant effect of treatment, albeit a small effect size [F_(1,67)_ = 7.283, *p* = 0.008, ηp^2^ = 0.053] with ELS animals sleeping more during playback than Control (CTR: 88.577 ± 25.047 s, ELS: 182.633 ± 24.760 s). An interaction between sex and treatment approached significance [Treatment/Sex: *F*_(1,67)_ = 3.088, *p* = 0.081]. Post-hoc comparisons revealed a significant effect of ELS in the amount of time female gerbils spent sleeping during sound playback, with Control females sleeping less than ELS females [CTR: 38.046 ± 38.011 s, ELS: 194.346 ± 35.556 s, one-way ANOVA, *F*_(1,58)_ = 6.798, *p* = 0.012, ηp^2^ = 0.105]. The ELS treatment did not appear to affect the amount of time male gerbils spent sleeping during playback [CTR: 139.107 ± 26.107 s, ELS: 171.610 ± 31.419 s, one-way ANOVA, *F*_(1,58)_ = 0.642, *p* = 0.426].

These data reveal no apparent effect of stimulus type or sex on level of arousal during playback. The ELS treatment, however, did significantly influence playback sleeping: ELS animals slept significantly longer than Control. While there was not an interaction effect of treatment and sex, the Control female gerbils slept significantly less than the ELS females. The extreme variability across animals in amount of time spent sleeping underlines the importance of measuring approach-related behavioral data as a proportion of time spent awake.

### 3.4. Locomotion during playback

One measure of interest in the vocalizations would be a reduction in locomotion during vocalization playback compared with silent baseline. Because vocalization valence may also affect locomotion, we first compared entry rates during Alarm Call and Contact Call playback. Because we found no difference between the stimulus types, we combined them in subsequent entry rate analyses [paired-samples *t*-test, t_(1,26)_ = 0.072, *p* > 0.20].

**FIGURE 4:**
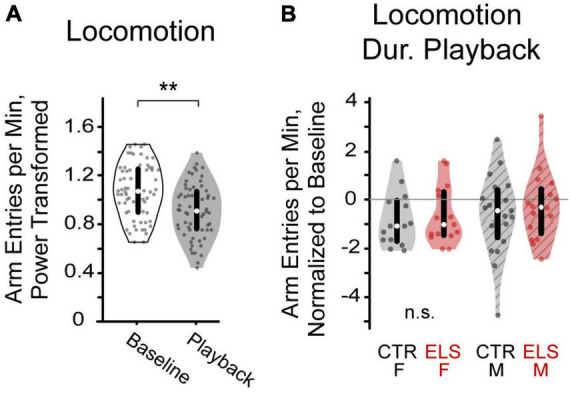
**(A)** Playback of vocalizations decreased locomotion in gerbils, as measured by arm entry rate. **(B)** After normalizing arm entry rate to baseline, neither early life stress (ELS) nor sex affected locomotion during vocalization playback **p < 0.001.

Paired-samples *t*-tests comparing average number of arm entries per minute during baseline and playback revealed a lower arm entry rate during the vocalization playback regardless of the vocalization stimulus [Figure 4A, Baseline: 1.085 ± 0.021 s, Playback: 0.902 ± 0.020 s, paired-t_(1,113)_ = 9.253, *p* < 0.001].

To determine any effects of treatment and sex on interest in the vocalizations, we also compared groups on the rate of locomotion during playback. To account for any differences in baseline locomotion, locomotion during playback was normalized as rate of locomotion during playback divided by rate of locomotion during baseline. No differences in normalized arm entry rate were found between the ELS and Control groups [Figure 4B, *F*_(1,62)_ = 0.561, *p* > 0.20], nor between females and males [*F*_(1,62)_ = 0.629, *p* > 0.20]. We found no interaction effects between treatment and sex [*F*_(1,62)_ = 0.060, *p* > 0.20].

In all, we found that animals reduce their locomotion rate during the vocalization playback relative to baseline, but the valence of the vocalization does not affect locomotion. Thus, playback locomotion, like baseline locomotion is not affected by treatment or sex.

### 3.5. Time spent approaching the sound source

Because previous studies have found differential responses to conspecific vocalizations based on sex of the listener (Ghasemahmad, 2020; Niemczura et al., 2020), the data were analyzed separately for males and females. The amount of time spent approaching the sound source was used to assess interest and investigation of vocalizations.

We implemented an approach metric by measuring the proportion of total time awake during playback and during silent baseline. This was necessary because there was great inter-subject variation in amount of time spent sleeping. Further, because gerbils displayed a wide inter-subject range of locomotion, we normalized this proportion to the individual’s proportion of time spent in the approach arms during silent baseline.

A two-way repeated measures ANOVA examined the within-subjects effect of stimulus type and between-subjects effects of treatment and sex on approach behavior. There was a trend for stimulus valence to affect approach behavior within animals, with the alarm stimulus eliciting approach but the contact stimulus eliciting no change in approach [Alarm: 0.327 ± 0.167; Contact: –0.001 ± 0.064; F_(1,25)_ = 2.647, *p* = 0.116, ηp^2^ = 0.096]. There were no between–subjects main effects of treatment [F_(1,25)_ = 0.173, *p* > 0.20]. A main effect of sex on approach behavior approached significance with females strongly approaching the stimulus and males showing modest approach behavior [Females: 0.304 ± 0.112; males: 0.022 ± 0.102; F_(1,25)_ = 3.468, *p* = 0.074, ηp^2^ = 0.122]. Finally, there was a significant interaction of treatment and sex [F_(1,25)_ = 4.962, *p* = 0.035, ηp^2^ = 0.166], which is explored below. Because of the near significant effect of stimulus valence on approach, and to simplify interpretation, we separated the alarm and contact call analyses.

#### 3.5.1. Alarm calls

**FIGURE 5:**
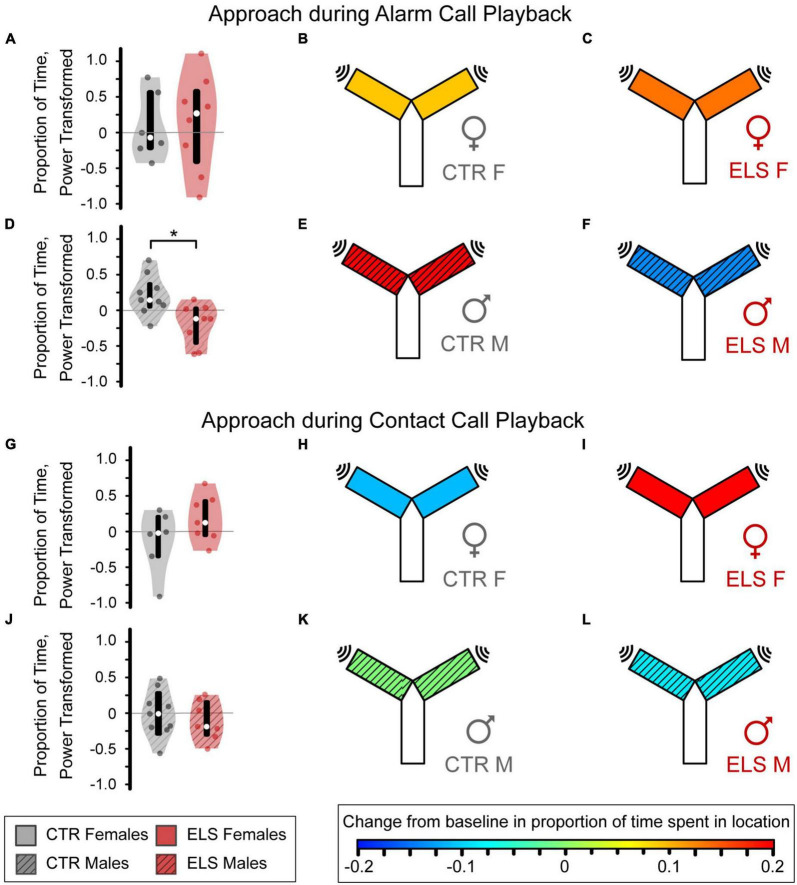
Early life stress (ELS) and sex influence approach response to alarm **(A–F)**, but not contact vocalizations **(G–L)**. Heatmap colors on the Y-maze schematic indicate the average amount of approach or avoidance of the sound source. **(A)** For the alarm vocalizations, no differences were seen between the Control **(B)** and ELS **(C)** female gerbils. **(D)** The Control males **(E)** approached the alarm vocalization stimulus while the ELS males **(F)** decreased their presence near the sound source. **(G)** For the contact vocalizations, ELS did not affect amount of time female gerbils spent near the contact call. **(H)** Control females avoided the call while ELS females **(I)** approached the source. **(J)** No differences were seen between Control **(K)** and ELS **(L)** male gerbils. Violin plots display the interquartile range (box), median (empty circle), and range, with a colored circle for each individual. *Signficance at *p* < 0.05.

Control females, early-life stress (ELS) females, and Control males increased time spent near the sound in response to alarm call playback, as indicated by warmer heatmap colors which represent a positive mean approach metric (Figures 5B, C, E). ELS treatment did not affect female alarm call approach [Figure 5A, Control female: 0.090 ± 0.243, ELS female: 0.136 ± 0.211, one-way ANOVA, F_(1,13)_ = 0.020, *p* > 0.20, ηp^2^ = 0.002]. In males, ELS significantly influenced the alarm call playback response [Figure 5D, F_(1,16)_ = 5.949, *p* = 0.028, ηp^2^ = 0.284] as ELS males had a negative mean approach metric, reflecting decreased time spent near the alarm call (Figures 5E, F, Control: 0.198 ± 0.094, ELS: –0.140 ± 0.102).

#### 3.5.2. Contact calls

Control female and early-life stress (ELS) male gerbils spent less time near the sound source during contact call playback than during baseline, as indicated by a negative mean approach metric and cooler heatmap colors (Figures 5H, L, Control female: –0.112 ± 0.181, ELS male: –0.089 ± 0.105). Conversely, contact call playback led to a great interest in the sound in ELS Females as indicated by a large, positive mean approach metric (Figure 5I, 0.201 ± 0.125). However, there was only a trend toward a difference between ELS and Control females [Figure 5G, F_(1,12)_ = 2.127, *p* = 0.173, ηp^2^ = 0.162]. The contact call did not influence Control males’ mean approach behavior (Figure 5K, Control male: –0.006 ± 0.109), which was not significantly different from ELS males [Figure 5J, F_(1,15)_ = 0.284, *p* > 0.20, ηp^2^ = 0.020]. Overall, our findings indicate that ELS reduces the approach response to alarm calls in male gerbils and may increase the approach responses to contact calls in female gerbils.

## 4. Discussion

Early-life stress (ELS) impairs the perception of temporally-varying auditory signals (Ye et al., 2022). Because temporal processing is critical for acoustic communication, we hypothesized that ELS impairs interpretation of, and behavioral responses to, vocalizations. To test this idea, we created an ethologically relevant Y-maze which allowed adolescent Mongolian gerbils to freely engage with two vocalization types of polar valence. Their behavioral responses indicate that the effect of ELS on vocalization salience is dependent on the valence of the signal and the listener’s sex.

### 4.1. Effects of ELS and sex on vocalization approach

For alarm calls, early-life stress (ELS) led to a reduction in approach in male gerbils but did not alter the response of female gerbils. This aligns with previous studies in our lab, which have shown that ELS gerbils learn aversive tasks faster than control animals, suggesting that they may be more motivated to avoid an aversive stimulus such as an alarm call that signals danger (Hardy et al., 2019). This sex difference is consistent with sex roles of wild Mongolian gerbils where males (more than females) patrol a colony’s territory for threats from intruders or predators (Agren et al., 1989b). There may be an innate instinct in Control male gerbils guiding them to investigate the potential threat suggested by the alarm call playback and ELS may interrupt this behavior.

Although there were no statistically significant differences across treatment groups during playback of the contact call, ELS females were the only group that approached the contact calls. The lack of approach in Control females and both groups of males suggests that the contact calls used here may not have served as the intended pro-social signal; contact calls can also be emitted during early stages of aggression (Ter-Mikaelian et al., 2012). The increased approach toward contact calls in ELS females may be related to impulsivity. While the effects of ELS on impulsive choice (difficulty delaying gratification) are mixed, the deleterious effects of ELS on impulsive action (the inadequate inhibition of a behavior) have been well-documented in both sexes of humans (Van Den Bergh et al., 2005; Sanchez and Bangasser, 2022) and rodent models (Lee et al., 2016; Murthy and Gould, 2018; Uekita et al., 2019). For example, chronic adolescent stress in rats leads to faster decision-making in response to ambiguous stimuli and shortens the latency to explore a novel object, two proxies for impulsivity (Chaby et al., 2013).

This increase in impulsive action has been attributed to ELS-induced neural differences in the medial PFC (mPFC) and anterior cingulate cortex (Liston et al., 2006; van Bodegom et al., 2017; Sanchez and Bangasser, 2022). Additionally, the lack of a similar effect in ELS male gerbils may be due to sex-differences in salience of the contact call-in their natural setting, female gerbils engage in significantly more social interactions than males (Yapa, 1994; Starkey et al., 2007; Hurtado-Parrado et al., 2015).

To our knowledge, only one other study has investigated behavioral responses to adult conspecific vocalizations in gerbils, and found that adult male gerbils avoid alarm calls and approach contact calls; females were not tested (Kobayasi et al., 2014). However, our adolescent male gerbils approached the alarm calls and did not change their behavior in response to contact calls. This may be attributable to developmental state: for example, relative to adulthood, male rats show a spike in novel object exploration rates (a proxy for impulsive actions) during mid-adolescence (Cyrenne and Brown, 2011). Thus, the approach/avoidance disparities across these two studies, and the reason our adolescents approached the alarm call, may arise from the widely known increase in impulsivity and exploration during adolescence when the mPFC is still maturing (Stansfield and Kirstein, 2006; Silvers et al., 2007; Ripke et al., 2012).

Also of note is that female gerbils in the wild are less likely to be on patrol for danger, but Control and ELS females approached the alarm call. This may be related to the confound of using an olfactory cue to provide context for the gerbils. Urine swabs from same-sex cage-mates were placed near the sound sources. This familiar smell of a close relative may have provided an extra incentive for the highly social females to approach the sound source despite being near an aversive stimulus, the alarm call. Indeed, olfactory cues change the extent of approach to communication sounds in a Y-maze from mice (Grimsley et al., 2013).

Further, the studies differed by both context in which the alarm call was emitted, and cadence in the contact calls. Kobayashi only used alarm calls produced by a single male after it was bitten, while our calls were from males and females, elicited through startling. Also, their contact call stimulus was a natural bout of contact calls, while we used individual syllables to match the alarm call, which is typically a single syllable. Sex differences in perceived vocalization salience are also seen in mice. A playback study similar to this study used a vocalization category associated with negative valence. In response to these negative calls, male mice had higher escape initiation and freezing behaviors than females (Ghasemahmad, 2020). This reinforces the importance of analyzing female and male data separately.

### 4.2. Effects of ELS and sex on non-approach behaviors

#### 4.2.1. Locomotion

One underlying non-auditory influence on vocalization approach behavior is locomotion. We can rule out any baseline differences in locomotion across treatment types or sex, as all groups showed similar average arm entry rates during baseline. Adult gerbils similarly lack sex differences in locomotion (Bridges and Starkey, 2004; but see Jaworska et al., 2008). Similar levels of baseline locomotion across the sexes have been found in both Control and ELS mice (Bath et al., 2017a). During playback, we saw a reduction in locomotion regardless of vocalization valence. While this could suggest that the animals were spending more time listening than exploring, the results are potentially confounded by the lengthy baseline period immediately preceding the playback, as exploration during baseline may have increased locomotion. As during baseline, there were also no effects of stress, sex, or vocalization valence on locomotion during playback.

#### 4.2.2. Cognition

While this study did not directly assess the effects of early-life stress (ELS) and sex on cognition, we measured the arm-entrance patterns during baseline as a proxy for attention and working memory, two aspects of cognition governed largely by PFC and hippocampus (Salmon et al., 1996). Sufficient attention and working memory are required for basic appetitive behaviors such as escape and finding food, as well as exploration, and such behaviors are considered motivating factors for animals in a Y-maze. In such an inescapable, un-baited maze, the “win-stay, lose-shift” strategy discourages animals from returning to recently visited less interesting locations (Richman et al., 1986). This strategy is dependent on an animal’s ability to orient itself in the maze and to remember which locations it visited most recently.

One measure of the ability to “lose-shift” is the rate of alternation errors, (i.e., re-entering the most- or second-most-recent arm). While we found no effect of ELS on the number of alternation errors, there was an effect of sex, with adolescent male gerbils making more errors than females. Similar results have been found in adult gerbils and golden hamsters (Hughes, 1989; Llorente et al., 2011). A higher error rate is typically interpreted as a cognitive impairment, based on decades of behavioral outcomes in gerbil ischemia research (Wappler et al., 2009). In the present study, we do not suggest that our adolescent males have *impaired* cognition relative to females, but simply that cognition between males and females differs. The sexes may vary in general preference for novelty, motivation to explore novel locations, habituation rates, or navigational strategies.

The second measure of an animal’s application of “lose-shift” is the spontaneous alternation (SA) rate (i.e., rate of entering an arm different from the previous two). Neither ELS nor sex affected SA rates in adolescent gerbils. The literature shows mixed effects of ELS on SA rates, a common obstacle in this field. The last measure of an animal’s application of “lose-shift” allows the SA rate of each sex and treatment group to be compared independently to chance level (0.5). Successful exploitation of “lose-shift” is reflected by an SA rate above chance levels (Richman et al., 1986). In the present study, SA rates in adolescent male gerbils were significantly below chance, while adolescent female gerbils performed at chance. Low SA rates reflect re-entering recently visited arms and signify that males are able to orient within an arena, have a sufficient capacity for spatial working memory to apply “lose-shift,” and preference familiarity. In the absence of any sex differences in sleep and locomotion, this male preference for familiarity cannot be accounted for by increased anxiety-related behaviors or activity levels.

Working memory and orientation abilities are not likely to cause the chance alternation in females because they made significantly fewer alternation errors than males and males were proficient in these two abilities. Incomplete habituation can also be ruled out as there were no sex differences in locomotion or anxiety-like behaviors. A more likely explanation is that these females were tested during an ontological transition period of novelty-seeking. Young rodents do not show the strong novelty preference seen in adults (Kirkby, 1967; Richman et al., 1986). Our P31–32 female gerbils may not yet have had the capacity to form a preference. Indeed, to the best of the authors’ knowledge, the female advantage in the alternation error rate (above) is the first report of any prepubescent sex differences in a spontaneous alternation measure in any species (Kirkby, 1967; Hughes, 1989; Thompson et al., 2005; Rodríguez et al., 2013; Shansky, 2018).

Taken together, our two measures of alternation suggest that the ELS and Control animals have similar cognitive abilities, indicating that differences in cognition are not likely to affect other measurements, though additional direct measures of cognition would strengthen this conclusion. Sex, however, may influence other outcomes as females performed at chance level, while males performed below chance and may be averse to exploring less-familiar areas.

#### 4.2.3. Arousal

High arousal is a defining characteristic of anxiety, and stress is known to increase arousal (De La Casa et al., 2016). Because sleep requires relaxation, animals tend not to sleep when highly aroused (Bangasser et al., 2018). There was an effect of ELS on the amount of time spent sleeping during playback, but not during baseline, which suggests that ELS may affect the level of arousal induced by conspecific vocalizations. ELS animals slept more than Control animals regardless of vocalization valence or sex, suggesting ELS influences arousal induced by communication sounds. Curiously, a previous study in adult gerbils found contradictory results from ours: sex, but not social isolation stress, affects sleeping (Rico et al., 2019). The significant difference in time each subject was awake was compensated for in all non-sleeping behavioral measures by measuring the behaviors as proportion of time awake rather than total time of the trial.

One limitation of the current study is the length of the baseline period directly before the playback. Relative to baseline, there was an overall increase in sleeping and reduction in locomotion during playback, suggesting potential fatigue or lack of interest. Lengthening the short habituation period on the day prior to the start of the experiment and shortening the pre-playback baseline period on the experimental days could alleviate this issue.

### 4.3. Implicated neural regions

#### 4.3.1. Auditory

A recent study from our lab found that early-life stress (ELS) in gerbils alters neural responses at multiple steps in the auditory pathway. Responses to temporally varying auditory stimuli were reduced in auditory brainstem response (ABR) waves I and II, indicating a deficit at the level of the auditory nerve and auditory brainstem. These changes were accompanied by deficits in behavioral detection of the stimuli (Ye et al., 2022). A similar deficit was seen in ABRs of adolescent humans from low socio-economic status (SES)-environments (Skoe et al., 2013), where low-SES is a proxy for stress. Other subcortical auditory regions show stress-induced dendritic atrophy after chronic adult stress in male rats, including the auditory midbrain and thalamus (a relay station from the midbrain to the auditory cortex (ACx) and/or to the limbic system via the basolateral amygdala) (Dagnino-Subiabre et al., 2005, 2009). While our lab’s data suggest the ACx is altered by ELS, the reduction in signal fidelity begins earlier in the auditory pathway, at the auditory nerve in our gerbil ELS model, and with inner hair cell atrophy in the cochlea of adult female rats with high corticosterone levels (Singer et al., 2018; Ye et al., 2022). However, a degraded signal representation in the auditory system cannot be the only factor in this experiment because the responses of the ELS animals depended both on the valence of the vocalization and the listener’s sex.

#### 4.3.2. Non-auditory

After information ascends through the auditory pathway to the auditory thalamus, it continues ascending to the ACx and/or projects to the limbic system, which in turn influences auditory perception via modulation of the ACx and auditory thalamus (Brudzynski, 2010). The limbic system, namely, the amygdala, is affected by ELS (Malter Cohen et al., 2013; Burgdorf et al., 2020) and plays a role in response to salient vocalizations (Grimsley et al., 2013). Specifically, the basolateral amygdala (BLA) has been implicated in behavioral responses to auditory stimuli in humans, rodents, and many other animals (Grimsley et al., 2013; Wenstrup et al., 2020). Another region that shapes auditory perception and is known to be affected by ELS is the PFC, which plays a vital role in attention, decision-making, and learning (Krugers et al., 2017; Kolk and Rakic, 2022). Both regions influence the auditory pathway via modulation of the auditory cortex and thalamus. For example, in mice, optogenetic stimulation of the orbitofrontal cortex evokes neural activity in the ACx and pairing this stimulation with tones alters ACx receptive fields (Winkowski et al., 2013, 2018).

### 4.4. Limitations

A clear limitation of the present study is that we cannot distinguish how much the ELS and sex effects arise from altered encoding of the auditory signal or from altered processing in neural regions involved in attending to and ascribing meaning to the vocalizations. Further, we cannot distinguish whether the perception of the acoustic signal or the behavioral response is altered. Although recent data from our lab have revealed an effect of ELS on neural encoding in the auditory system, as well as deficits in a non-attentive auditory perceptual task (Ye et al., 2022), we cannot rule out physiological differences excluding the rate of locomotion.

Future studies on the effects of ELS on auditory-related behavior in Mongolian gerbils should include more direct measures of cognition (e.g., attention, learning, and problem solving), as well as a proxy for *anxiety* (including stereotypies such as compulsive digging), rather than the proxy for *arousal* used in the present study.

#### 4.4.1. The analysis time window may influence findings in behavioral studies

Vocalization playback studies frequently measure behavior for >3 min after stimulus playback in order to capture all relevant behaviors (Wöhr and Schwarting, 2007; Chabout et al., 2012; Grimsley et al., 2013; Fernández-Vargas and Johnston, 2015). Here, we chose to measure behavior during the 1-min vocalization playback and the 5 min silent inter-trial-interval because external stimuli alter gerbil behavior for several minutes. For example, gerbils hide in burrows after an alarm call given in a semi-naturalistic setting for ∼5–10 min and continue to show alert behavior when they re-emerge (Yapa, 1994; Randall et al., 2002). While we provided no burrows for hiding, we wanted to ensure our sampling window was wide enough to catch all relevant behaviors (Yapa, 1994; Ter-Mikaelian et al., 2012). Further, a common gerbils response to alarm call playback is to drum (thump their feet), which can persist for up to 10 min (Ter-Mikaelian et al., 2013). Gerbil and other rodent behavioral responses to non-auditory stimuli also show latencies of up to 10 min (Bialy et al., 2019; Furuyama et al., 2022).

Stressful stimuli induce both a quick stress response (i.e., fight or flight) and a slow stress response (Wright et al., 2007; McEwen et al., 2015; Wilson et al., 2015). The slow stress response induces an increase in HPA-axis activity, that eventually (minutes to hours) leads to a homeostatic change, including an increase in CORT levels (Jafari et al., 2017). The altered CORT levels may be partially responsible for time-delayed behavioral changes. The quick stress response, mediated by the sympathetic nervous system, triggers immediate release of epinephrine and norepinephrine (Eilam et al., 1999; Jafari et al., 2017). The onset of the response is abrupt, but the offset is gradual, necessitating an extended time window of analysis. For example, norepinephrine levels in the auditory cortex peak around 3–4 min after the stimulus (Pérez-Valenzuela et al., 2016). Thus, it is important to choose a time window that encompasses delayed behavioral responses due to gradual physiological changes, which should occur differentially in ELS animals. Future studies should analyze behaviors during multiple time windows to gain information about the change in behavioral response to a stimulus over time, encompassing quick and slow stress responses.

#### 4.4.2. The vocalization stimuli did not strongly affect the control animals

The impetus for this study was to investigate the effects of early-life stress (ELS) on approach to two particular types of vocalizations. While this objective was achieved, the approach response of Control animals to the vocalizations was minimal. The Control animals responded less to the vocalizations than ELS animals, suggesting a difference in response motivation, perhaps driven by differences in perceived salience of the vocalizations. The increase in amygdalar activity often seen in ELS animals is one possible cause for salience differences; the ELS amygdala may ascribe a stronger emotional relevance to the stimulus than the Control amygdala.

There are many other reasons Control animals may not have been as motivated by our stimuli, including lower attention due to the long habituation or due to hyperactivity during adolescence, the artificial rhythms of our call bouts, or ineffectiveness of the olfactory cue, which is necessary for some gerbil approach behaviors. While non-stressed gerbils in a previous study did respond strongly to an aversive call (a combination of alarm syllable and two other syllables), the context during which this vocalization was emitted was after one animal violently attacked another (Kobayasi et al., 2014). To better understand the response of non-stressed gerbils to vocalizations, future studies should include a wider range of behaviors, including head orientation, rearing, freezing, flinching, and grooming.

## 5. Conclusion and implications

Early life stress has widespread effects on physiology and behavior. Its influence on brain regions governing cognition, emotional regulation, and memory have been well-studied, but we know very little about the effect of ELS on the auditory pathway or auditory perception. The finding that ELS influences behavioral responses to ethologically relevant sounds in a sex-specific manner provides valuable information on auditory processing. The tests in the present study on behaviors related to cognition, anxiety, and memory do not support a higher-level influence on auditory processing, but ruling out top-down influences goes beyond the scope of this study. However, another study from our lab found a discernable bottom-up influence of ELS, leading to poorer detection of short gaps in sound in a pre-attentive behavioral measure (pre-pulse inhibition of the acoustic startle response), along with impaired encoding of gaps in the auditory pathway (Ye et al., 2022). As it is likely that our results here are a combination of top-down and bottom-up influences, more direct testing of this emerging model of ELS is needed.

The present study is a crucial early step toward lessening the impact of communication disorders, which affect almost 8% of US children aged 3–17. These communication disorders can be exacerbated by hearing difficulties, which impact almost 15% of US children aged 6–19 (Niskar et al., 1998; Black et al., 2015). Our study suggests that early-life stress affects interpretation of vocal communication in adolescent Mongolian gerbils, likely driven in part by altered auditory temporal processing (Ye et al., 2022). Such communication-related impairments in early adolescent humans could be detrimental in various settings including hearing in noisy classrooms and understanding emotional content during social interactions.

## Data availability statement

The raw data supporting the conclusions of this article will be made available by the authors, without undue reservation.

## Ethics statement

This animal study was reviewed and approved by the Institutional Animal Care and Use Committee, Northeast Ohio Medical University.

## Author contributions

KH and MR designed the experiments and designed the analysis. DH and KH collected the data. KH analysed the data and wrote the manuscript. MR edited the manuscript. All authors contributed to the article and approved the submitted version.
